# The Effects of Depression and Anti-Depressants on Quality of Life After Breast Reconstruction: A Post-Hoc Analysis

**DOI:** 10.7759/cureus.18675

**Published:** 2021-10-11

**Authors:** Kevin M Klifto, Faraah N Bekheet, Michele A Manahan, Kristen P Broderick, Damon S Cooney, Gedge D Rosson, Carisa M Cooney

**Affiliations:** 1 Plastic and Reconstructive Surgery, University of Missouri, Columbia, USA; 2 Department of Plastic and Reconstructive Surgery, Johns Hopkins University School of Medicine, Baltimore, USA; 3 Department of Plastic and Reconstructive Surgery, Johns Hopkins Health System, Baltimore, USA

**Keywords:** patient-reported outcome measures, psychiatry and mental health, quality of life (qol), breast plastic surgery, plastic and reconstructive surgery, breast disease, breast cancer, depression, anti-depressants, breast reconstruction

## Abstract

Background

A personal history of depression prior to breast cancer diagnosis and its effect on post-diagnosis quality of life (QOL) in women undergoing breast reconstruction is relatively unknown. We performed the current study to determine if depression alters QOL for patients who undergo breast reconstruction by assessing the pre-to-post-operative change in patient-reported BREAST-Q scores.

Methodology

We conducted a single-center, post-hoc analysis of 300 patients with completed BREAST-Q data who underwent breast reconstruction from November 2013 to November 2016 following a diagnosis of breast cancer. Patients completed the BREAST-Q at four time points: pre-operatively, six weeks following tissue expander (TE) insertion for patients undergoing staged reconstruction, and six and 12 months following the final reconstruction. Medical records were reviewed to identify patients who had a pre-cancer diagnosis of clinical depression and/or anti-depressant medication use. BREAST-Q scores were compared between groups and within groups. Groups compared were the depression (n = 50) and no depression (n = 250) patients, along with anti-depressant (n = 36) and no anti-depressant (n = 14) use in the depression group.

Results

Within-group Sexual Well-being scores at the six-week post-TE follow-up for patients in the depression group (median = 37, interquartile range [IQR] = 25-47) were significantly lower (p < 0.01) than the scores for patients in the no depression group (median = 47, IQR = 39-60). There were no statistically significant differences in BREAST-Q scores in other domains. When compared to patients diagnosed with depression who were not taking anti-depressants, anti-depressant medication use did not result in statistically significant higher BREAST-Q scores, although Satisfaction With Breasts six months post-operatively, Psychosocial Well-being at six weeks post-TE, Sexual Well-being at six weeks post-TE and six months post-operatively were clinically higher in patients taking anti-depressants for depression.

Conclusions

Patients with a diagnosis of depression prior to breast cancer had lower BREAST-Q Sexual Well-being scores in the six-week TE group with or without anti-depressant medication. Patients with a pre-cancer diagnosis of depression considering TEs may benefit from additional counseling prior to breast reconstruction or electing a different method of breast reconstruction. Anti-depressant medications may provide clinically higher BREAST-Q scores in patients with a pre-cancer diagnosis of depression. Adding an anti-depressant medication to a patient’s treatment plan may provide additional benefits. Larger samples are required to properly determine the impact of anti-depressant medications on BREAST-Q scores in patients with a pre-cancer diagnosis of depression.

## Introduction

In 2018, the Centers for Disease Control and Prevention reported that 8.1% of all U.S. adults aged 20 and older had a diagnosis of depression, with women being nearly two times more likely than men to be diagnosed [1]. Diagnosis requires depressed mood or loss of interest in five of nine criteria for a minimum of two weeks. Importantly, this diagnosis must not include symptoms attributable to the physiological effects of another medical condition or substance use [2,3].

Breast cancer is the most prevalent cancer among women and the second leading cause of cancer mortality in the United States. An estimated one out of every eight U.S. women will be diagnosed during their lifetime [4,5]. Breast cancer and its corresponding treatment have an overall negative physical and psychological effect on patients [6]. Quality of life (QOL), a term used to describe the personal well-being and life satisfaction of individuals, can be negatively impacted after breast surgery due to changes in self-perception of physical appearance [7]. These negative effects often contribute to depression [8]. While the American Cancer Society states that most patients and families are faced with some degree of depression when cancer becomes a part of their lives, six pharmacologic anti-depressant treatments have been shown to significantly improve depression and QOL in breast cancer patients [9,10].

Women with a history of depression have been shown to have a higher risk for recurrence of depression following breast cancer diagnosis [2]. However, few studies have assessed longitudinal QOL in breast cancer patients with a pre-cancer history of depression [11,12]. One study using the Short Form Health Survey 36 (SF-36) found that patients with a pre-cancer diagnosis of depression had lower QOL scores than women newly diagnosed with depression following a cancer diagnosis [11]. A similar study reported that, compared to pre-cancer scores, depressive symptoms increased 20% at 0-6 months and 12.9% at 6-12 months following breast cancer diagnosis in patients with a history of depression [12]. These patients’ physical function and mental health also decreased, and the authors found that patient-reported QOL remained significantly lower 10 years post-breast cancer diagnosis [12]. Additionally, a pre-cancer diagnosis of depression was associated with increased all-cause mortality, even when treated with anti-depressant medication [13,14].

To our knowledge, no study has examined the differences in pre- and post-operative patient-reported outcomes (PROs) in patients undergoing breast reconstruction with a pre-cancer diagnosis of depression. We performed the current study to determine if BREAST-Q scores differ between patients with and without a pre-cancer diagnosis of depression by looking at PROs at various time points throughout the reconstruction process. Additionally, we examined if anti-depressant medication use in patients diagnosed with depression impacted patient-reported QOL and satisfaction reported in the BREAST-Q.

## Materials and methods

Study design

This study was approved by the institutional review board at The Johns Hopkins Hospital (IRB00039871). The STROBE guidelines are adhered to throughout this paper. We performed a post-hoc analysis of a prospectively collected cohort of 300 patients from a single institution who underwent breast reconstruction following a diagnosis of breast cancer with completed BREAST-Qs at designated time points from November 2013 to November 2016. Patients were included if they were ≥18 years of age; underwent immediate or staged (tissue expander [TE] insertion) or delayed (mastectomy followed by reconstruction on a later date) breast reconstruction; and had completed BREAST-Qs at the designated time points: pre-operatively (baseline), six weeks following TE insertion (only for staged reconstruction patients), and six and twelve months following final reconstruction. Included patients in the study may have received implant-based or autologous breast reconstruction. We reviewed patient medical records to identify individuals with a pre-cancer diagnosis of clinical depression and/or anti-depressant medication use indicated for the treatment of depression.

We categorized patients as having depression if pre-breast cancer Diagnostic and Statistical Manual of Mental Disorders, fifth edition (DSM V) criteria clinical diagnosis of depression could be identified in their medical records. We defined no depression as the absence of this diagnosis and no history of taking anti-depressant medication for the indication of depression prior to breast cancer diagnosis. We further subdivided patients in the depression group into one of two groups: those who were (medication use) and those who were not (medication non-use) taking anti-depressant medications at the time of pre-cancer diagnosis and throughout the reconstruction process, and recorded anti-depressant medication use as relevant. Daily medication adherence was confirmed by all patients included in the study. Psychotherapy and other non-medication therapies were not evaluated in this study. We excluded patients taking an anti-depressant medication in the absence of a clinical diagnosis of depression from the depression group.

We assessed patient QOL and satisfaction using five domains within the BREAST-Q breast reconstruction module (Appendix): (1) Satisfaction With Breasts; (2) Psychosocial Well-being; (3) Sexual Well-being; (4) Physical Well-being: Chest; and (5) Physical Well-being: Abdomen. For each domain, items were summed and converted to a 0-100 scale, with greater values indicating higher satisfaction and better QOL [15].

Outcomes analyzed

Our primary outcome of interest was the difference in BREAST-Q reconstruction module scores between patients with depression and with no depression at our four study time points: (1) pre-operatively (baseline); (2) six weeks post-TE insertion (for staged reconstruction), (3) six months after final reconstruction, and (4) 12 months after final reconstruction. Our secondary outcome of interest was the difference in BREAST-Q scores between depressed patients with medication use and depressed patients with medication non-use.

Data management and statistical analysis

Patient and BREAST-Q data were prospectively entered into and managed using the Research Electronic Database Capture (REDCap, version 8.3.1 2018, Vanderbilt University, Nashville, TN, USA). We performed descriptive statistics to compare patient demographics. Categorical variables and continuous variables were compared using the Fischer’s Exact test and the Mann-Whitney U test, respectively. We reported medians and interquartile ranges (IQRs) for BREAST-Q scores using the Mann-Whitney U test to compare the depression and no depression patient groups. We used the same statistical guidelines for the subgroup analysis within the depression group based on medication use/non-use. We depicted changes in scores within the depression, no depression, medication use, and medication non-use groups from pre-operative to the four different time points during mastectomy and reconstruction using longitudinal graphs. Statistical significance was observed at p < 0.05. Clinical significance was determined by a minimal important difference (MID) of 0.5 the standard deviation (SD) between median BREAST-Q scores [16]. All statistical analysis was performed using Stata (v13.0, StataCorp, College Station, Texas, USA).

## Results

Demographics

Patient demographic variables are summarized in Table 1.

**Table 1:** Patient demographics. IQR: interquartile range; BMI: body mass index; COPD: chronic obstructive pulmonary disease; DM: diabetes mellitus; CPM: contralateral prophylactic mastectomy; BPM: bilateral prophylactic mastectomy *Statistically significant differences in comparisons

Variables		No depression	Depression	P-value
Sample size, n		250	50	
Age, median (IQR)		49.6 (43–57)	51.4 (42–56)	0.74
BMI, median (IQR)		25.7 (22–31)	26.3 (22–30)	0.58
Race				0.23
	Unknown	4 (1.6%)	0 (0%)	
	White	184 (74%)	43 (86%)	
	Black	39 (16%)	6 (12%)	
	Other	23 (9%)	1 (2%)	
Smoking		10 (4%)	7 (14%)	0.013*
Comorbidities	Overall	83 (33%)	15 (30%)	0.74
	COPD	1 (0.4%)	1 (2%)	0.31
	Hyperlipidemia	5 (2%)	0 (0%)	0.59
	Hypertension	60 (24%)	11 (22%)	0.86
	Type 1 DM	2 (0.8%)	1 (2%)	0.42
	Type 2 DM	6 (2%)	0 (0%)	0.59
	Other pulmonary disorder	4 (1.6%)	0 (0%)	0.99
Indication				0.82
	Therapeutic	162 (65%)	31 (62%)	
	CPM	70 (28%)	16 (32%)	
	BPM	18 (7%)	3 (6%)	
Laterality				0.61
	Unknown	1 (0.4%)	0 (0%)	
	Unilateral	108 (43%)	19 (38%)	
	Bilateral	141 (56%)	31 (62%)	
Reconstruction type				0.10
	Unknown	1 (0.4%)	0 (0%)	
	Immediate	29 (12%)	12 (24%)	
	Staged	173 (69%)	32 (64%)	
	Delayed	47 (19%)	6 (12%)	
Complications		102 (41%)	28 (56%)	0.060

A total of 300 patients were eligible for study inclusion having completed BREAST-Qs at the pre-operative and six- and 12-month post-operative time points. Of these 300 patients, 50 (17%) had depression and 250 (83%) had no depression. The only significant difference between the depression and no depression groups was in the percentage of smokers (14% in depression and 4.1% in no depression groups; p = 0.013). Reconstruction type did not differ significantly between groups, with 205 (68%) patients undergoing staged reconstruction involving TE placement. Of these, 32 (16%) had a pre-cancer history of depression and 173 (84%) had none; all of these patients completed BREAST-Qs for the six-week post-TE time point.

Within the depression group (n = 50), 36 (72%) patients were and 14 (28%) patients were not taking anti-depressant medications from the time before a diagnosis of breast cancer throughout the study. There were no significant demographic differences between groups based on medication use/non-use.

Anti-depressant medications

Five classes of anti-depressant medications were used by patients in the depression group (Table 2).

**Table 2:** Anti-depressant medications used by patients with a pre-cancer diagnosis of depression. SSRI: selective serotonin reuptake inhibitor; SNRI: selective serotonin and norepinephrine reuptake inhibitor; TCA: tricyclic anti-depressants; MAOI: monoamine oxidase inhibitor

Medication class	Medication name	Number of patients (%)
SSRI	Sertraline	6 (17)
	Escitalopram	8 (22)
	Citalopram	2 (5)
	Fluoxetine	2 (5)
	Paroxetine	1 (3)
SNRI	Duloxetine	6 (17)
	Venlafaxine	2 (5)
TCA	Doxepin	1 (3)
MAOI	Tranylcypromine	1 (3)
Miscellaneous	Bupropion	2 (6)
	Mirtazapine	2 (5)
Combinations	Amitryptaline + sertraline	1 (3)
	Sertraline + bupropion	1 (3)
	Bupropion + fluoxetine	1 (3)

These included 22 selective serotonin reuptake inhibitors (SSRI), eight selective serotonin and norepinephrine reuptake inhibitors (SNRI), two tricyclic anti-depressants (TCA), one monoamine oxidase inhibitors (MAOI), six miscellaneous classes, and three combinations of more than one class. SSRIs were observed as the most frequently used class of anti-depressant medications (n = 22, 61%). Among the 22 SSRIs, escitalopram (n = 8, 22%) and sertraline (n = 8, 22%) were most frequently used. Medications were further categorized as those causing sexual dysfunction (n = 30, 83%) and non-dysfunction (n = 6, 17%) to explore the impact of medication-induced sexual dysfunction on BREAST-Q Sexual Well-being scores.

BREAST-Q scores

We found no statistically significant differences in pre-operative baseline BREAST-Q scores between the depression and no depression groups across all five domains (Table 3). However, the median baseline scores for Physical Well-being: Abdomen demonstrated a clinically significant difference with the depression group having a lower median baseline score (depression group median = 83, IQR = 83-100 versus no depression group median = 100, IQR = 83-100).

**Table 3:** Between-group comparisons of patients with (“Depression”) and without (“No Depression”) a pre-cancer diagnosis of depression at four time points. TE: tissue expander; IQR: interquartile range; SD: standard deviation; pre-op: pre-operative Mann-Whitney U test. Statistical significance was observed at p < 0.05 *Statistically significant differences in comparisons. Clinically significant differences were >0.5 SD; ^+^clinically significant differences in comparisons

Domain	Time point	No depression (n = 250)		Depression (n = 50)		P-value
		Median (IQR)	0.5 SD	Median (IQR)	0.5 SD	
Satisfaction With Breasts	Pre-op	53 (38–70)	13	53 (38–70)	13	0.88
	6 weeks post-TE	49 (40–60)	9	45 (40–48)	4.5	0.08
	6 months post-op	65 (55–78)	9	62 (52–85)	10.5	0.77
	12 months post-op	65 (53–77)	10	67 (53–81)	10	0.92
Psychosocial Well-being	Pre-op	67 (57–86)	10	66 (57–86)	9.5	0.53
	6 weeks post-TE	63 (51–82)	10	60 (43–72)	10	0.14
	6 months post-op	79 (63–100)	10.5	79 (57–86)	9.5	0.21
	12 months post-op	79 (62–100)	10.5	79 (55–92)	11.5	0.33
Sexual Well-being	Pre-op	57 (43–66)	11	54 (43–66)	10.5	0.68
	6 weeks post-TE	47 (39–60)	12	37 (25–47)	9	<0.001*
	6 months post-op	57 (43–75)	12.5	52 (43–69)	12	0.46
	12 months post-op	57 (46–72)	12	51 (38–71)	13	0.11
Physical Well-being: Chest	Pre-op	81 (68–91)	8	81 (68–91)	8	0.54
	6 weeks post-TE	71 (63–85)	7.5	71 (63–79)	6	0.42
	6 months post-op	77 (66–90)	8	77 (71–91)	7.5	0.32
	12 months post-op	81 (67–91)	8	81 (66–91)	9	0.93
Physical Well-being: Abdomen	Pre-op	100 (83–100)	7	83 (83–100)	7.5	0.55^+^
	6 weeks post-TE	76 (56–92)	10	---	---	---
	6 months post-op	79 (70–100)	11.5	79 (79–89)	9.5	0.54
	12 months post-op	89 (70–100)	10	89 (70–100)	10	0.79

**Figure 1:**
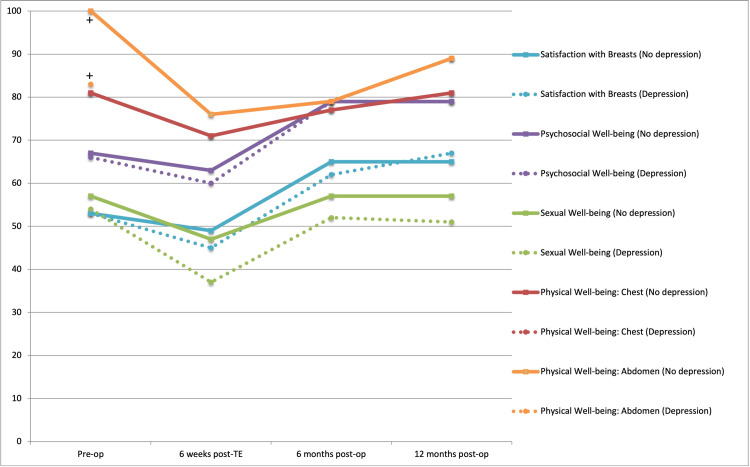
Differences in median BREAST-Q scores for patients with (“Depression”) and without (“No depression”) a pre-cancer diagnosis of depression at four time points. Patients with depression are represented by dotted lines and patients with no depression by solid lines. For the domains Psychosocial Well-being, Physical Well-being: Chest, and Physical Well-being: Abdomen, median BREAST-Q scores for both groups are nearly identical and overlap, appearing as a single solid line. No statistically significant differences were found. A clinically significant difference (+) [difference >0.5 standard deviation (SD)] between the Depression and No Depression groups was observed in Pre-operative Physical Well-being: Abdomen scores.

Figure 1 displays comparisons of BREAST-Q scores between the depression and no depression groups at the four time points. Scores were significantly lower for patients in the depression group (n = 32, median = 37, IQR = 25-47) than the no depression group (n = 173, median = 47, IQR = 39-60; p < 0.001) at the six-week post-TE placement time point for Sexual Well-being. Both groups demonstrated significant within-group BREAST-Q score decreases from the pre-operative to the six-week post-TE placement time point (Table 4).

**Table 4:** Within-group comparisons of patients with (“Depression”) and without (“No Depression”) a pre-cancer diagnosis of depression at four time points. TE: tissue expander; IQR: interquartile range; pre-op: pre-operative Differences in BREAST-Q scores were assessed between patients’ pre-op baseline and at the six-week post-TE placement, six months after final reconstruction, and 12 months after final reconstruction time points. Mann-Whitney U test. Statistical significance was observed at p<0.05; *statistically significant differences in comparisons.

Comparison of six-week post-TE with pre-op BREAST-Q scores					
Domain	Time point	No depression (n = 250)	P-value	Depression (n = 50)	P-value
		Median (IQR)		Median (IQR)	
Satisfaction With Breasts	Pre-op	53 (38–70)	<0.001*	53 (37–70)	0.006*
	6-week post-TE	49 (40–60)		45 (40–48)	
Psychosocial Well-being	Pre-op	67 (57–86)	0.01*	66 (57–86)	0.045*
	6-week post-TE	63 (51–82)		60 (43–72)	
Sexual Well-being	Pre-op	57 (43–66)	<0.001*	54 (43–63)	0.001*
	6-week post-TE	47 (39–60)		37 (25–47)	
Physical Well-being: Chest	Pre-op	81 (68–91)	<0.001*	81 (68–91)	<0.001*
	6-week post-TE	71 (63–85)		71 (63–79)	
Physical Well-being: Abdomen	Pre-op	100 (83–100)	0.01*	83 (83–100)	-
	6-week post-TE	76 (56–92)		-	
Comparison of six-month post reconstruction with pre-op BREAST-Q scores					
Domain	Time point	No depression (n = 250)	P-value	Depression (n = 50)	P-value
		Median (IQR)		Median (IQR)	
Satisfaction With Breasts	Pre-op	53 (38–70)	<0.001*	53 (37–70)	0.161
	6 months	65 (55–78)		64 (52–85)	
Psychosocial Well-being	Pre-op	67 (57–86)	<0.001*	66 (57–86)	0.883
	6 months	79 (62–100)		79 (57–86)	
Sexual Well-being	Pre-op	57 (43–66)	0.394	54 (43–63)	0.657
	6 months	57 (43–76)		52 (42–71)	
Physical Well-being: Chest	Pre-op	81 (68–91)	0.006*	81 (68–91)	0.651
	6 months	77 (66–91)		77 (71–91)	
Physical Well-being: Abdomen	Pre-op	100 (83–100)	<0.001*	83 (83–100)	0.296
	6 months	79 (70–100)		79 (75–89)	
Comparison of 12-month post reconstruction with pre-op BREAST-Q scores					
Domain	Time point	No depression (n = 250)	P-value	Depression (n = 50)	P-value
		Median (IQR)		Median (IQR)	
Satisfaction With Breasts	Pre-op	53 (38–70)	<0.001*	53 (37–70)	0.017*
	12 months	65 (53–77)		67 (53–81)	
Psychosocial Well-being	Pre-op	67 (57–86)	<0.001*	66 (57–86)	0.213
	12 months	79 (62–100)		79 (55–92)	
Sexual Well-being	Pre-op	57 (43–66)	0.124	54 (43–63)	0.816
	12 months	57 (46–72)		51 (38–71)	
Physical Well-being: Chest	Pre-op	81 (68–91)	0.986	81 (68–91)	0.276
	12 months	81 (67–91)		81 (66–91)	
Physical Well-being: Abdomen	Pre-op	100 (83–100)	<0.001*	83 (83–100)	0.127
	12 months	89 (70–100)		89 (70–100)	

The no depression group demonstrated significant within-group from pre-operative to six-month post-final reconstruction increases for Satisfaction With Breasts (median = 53, IQR = 38-70 versus median = 65, IQR = 55-78; p < 0.001), Psychosocial Well-being (median = 67, IQR = 57-86 versus median = 79, IQR = 62-100; p < 0.001), and decreases for Physical Well-being: Chest (median = 81, IQR = 68-91 versus median = 77, IQR = 66-91; p = 0.006), and Physical Well-being: Abdomen (median = 100, IQR = 83-100 versus median = 79, IQR = 70-100, p < 0.001). The no depression group demonstrated significant within-group from pre-operative to 12-month post-final reconstruction increases for Satisfaction With Breasts (median = 53, IQR = 38-70 versus median = 65, IQR = 53-77; p < 0.001), Psychosocial Well-being (median = 67, IQR = 57-86 versus median = 79, IQR = 62-100; p < 0.001), and decreases for Physical Well-being: Abdomen (median = 100, IQR = 83-100 versus median = 89, IQR = 70-100; p < 0.001). Within-group Physical Well-being: Abdomen scores increased for both the depression and no depression groups from six to 12 months after final reconstruction time points.

**Figure 2:**
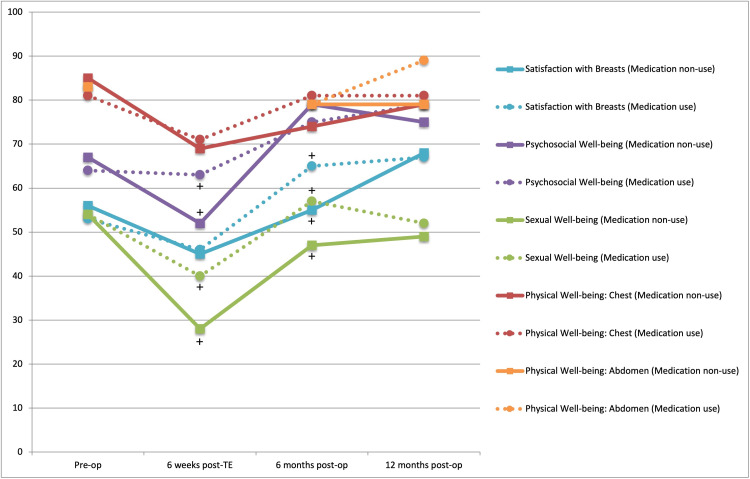
Differences in median BREAST-Q scores for patients with a pre-cancer diagnosis of depression who are and are not using anti-depressant medications at four time points. TE: tissue expander; SD: standard deviation Medication use is represented by dotted lines and medication non-use by solid lines. No statistically significant differences were found. Clinically significant differences (+) (differences >0.5 SD) between patients using and not using anti-depressants were observed in Psychosocial Well-being and Sexual Well-being at the six-week post-TE time point, and in Satisfaction With Breasts and Sexual Well-being at the six months post-operative time point.

Subgroup analysis of the depression group comparing medication non-use (n = 14, 28%) with medication use (n = 36, 72%) did not show any statistically significant between-group differences in BREAST-Q scores across any domains (Figure 2; Table 5). Two time points over three BREAST-Q domains demonstrated clinically significantly lower between-group scores in the medication non-use group: at the six-week post-TE time point, Psychosocial Well-being (median = 52, IQR = 38-76 versus median = 63, IQR = 50-73; delta = 11) and Sexual Well-being (median = 28, IQR = 25-41 versus median = 40, IQR = 20-49; delta = 12), and at the 6-month post-operative time point Satisfaction With Breasts (median = 55, IQR = 46-96 versus median = 65, IQR = 54-85; delta = 10) and Sexual Well-being (median = 47, IQR = 32-80 versus median = 57, IQR = 45-85; delta = 10) (medication users versus medication non-users, respectively) (Figure 2; Table 5).

**Table 5:** Between-group comparisons of patients with a pre-cancer diagnosis of depression who are and are not using anti-depressant medications at four time points. TE: tissue expander; IQR: interquartile range; SD: standard deviation; pre-op: pre-operative Mann-Whitney U test. Statistical significance was observed at p < 0.05; *statistically significant differences in comparisons. Clinically significant differences were >0.5 SD; ^+^clinically significant differences in comparisons.

Domain	Time point	Medication non-use (n = 14)		Medication use (n = 36)		P-value
		Median (IQR)	0.5 SD	Median (IQR)	0.5 SD	
Satisfaction With Breasts	Pre-op	56 (46–65)	11.5	53 (33–77)	13.5	0.80
	6 weeks post-TE	45 (37–48)	5	46 (40–49)	4.5	0.67
	6 months post-op	55 (46–96)	12.5	65 (54–85)	10	0.68^+^
	12 months post-op	68 (54–85)	8.5	67 (50–80)	11.5	0.53
Psychosocial Well-being	Pre-op	67 (60–75)	7	64 (54–91)	10	0.97
	6 weeks post-TE	52 (38–76)	11	63 (50–73)	8.5	0.61^+^
	6 months post-op	79 (51–82 )	8.5	75 (59–100)	10	0.50
	12 months post-op	75 (57–86)	9	79 (52–98)	12.5	0.65
Sexual Well-being	Pre-op	54 (38-65)	10	54 (48-63)	11	0.76
	6 weeks post-TE	28 (25–41)	4.5	40 (20–49)	10.5	0.34^+^
	6 months post-op	47 (32–80)	15.5	57 (45–68)	10.5	0.51^+^
	12 months post-op	49 (39–66)	12.5	52 (38–72)	13	0.73
Physical Well-being: Chest	Pre-op	85 (72–96)	7	81 (68–91)	8.5	0.48
	6 weeks post-TE	69 (61–77)	4.5	71 (63–81)	6.5	0.53
	6 months post-op	74 (67–88)	5.5	81 (70–93)	8.5	0.27
	12 months post-op	79 (65–91)	7.5	81 (68–91)	10	0.79
Physical Well-being: Abdomen	Pre-op	83 (83–100)	6	83 (83–100)	8.5	0.99
	6 weeks post-TE	---	---	---	---	---
	6 months post-op	79 (61–89)	9	79 (79–92)	10	0.56
	12 months post-op	79 (60–89)	8.5	89 (70–100)	10.5	0.30

The medication use group demonstrated a significant within-group decrease from the pre-operative to six-week post-TE placement for Satisfaction With Breasts (median = 53, IQR = 33-77 versus median = 46, IQR = 40-49, p = 0.029), Sexual Well-being (median = 54, IQR = 48-63 versus median = 40, IQR = 20-49, p = 0.001), and Physical Well-being: Chest (median = 81, IQR = 68-91 versus median = 71, IQR = 63-81; p = 0.001) (Table 6).

**Table 6:** Within-group comparisons of patients with a pre-cancer diagnosis of depression who are and are not using anti-depressant medications at four time points. TE: tissue expander, IQR: interquartile range; pre-op: pre-operative Differences in BREAST-Q scores were assessed between patients’ pre-operative baseline and at the six-week post-TE placement, six months after final reconstruction, and 12 months after final reconstruction time points. Mann-Whitney U test. Statistical significance was observed at p < 0.05; *statistically significant differences in comparisons.

Comparison of six-week post-TE with pre-op BREAST-Q scores					
Domain	Time point	Non-use (n = 14)	P-value	Use (n = 36)	P-value
		Median (IQR)		Median (IQR)	
Satisfaction With Breasts	Pre-op	56 (46–65)	0.116	53 (33–77)	0.029
	6-week post-TE	45 (37–48)		46 (40–49)	
Psychosocial Well-being	Pre-op	67 (60–75)	0.172	64 (54–91)	0.154
	6-week post-TE	52 (38–76)		63 (50–73)	
Sexual Well-being	Pre-op	54 (38–65)	0.225	54 (48–63)	0.001*
	6-week post-TE	28 (25–41)		40 (20–49)	
Physical Well-being: Chest	Pre-op	85 (72–96)	0.116	81 (68–91)	0.001*
	6-week post-TE	69 (61–77)		71 (63–81)	
Physical Well-being: Abdomen	Pre-op	83 (83–100)	---	83 (83–100)	---
	6-week post-TE	---		---	
Comparison of six-month post-reconstruction with pre-op BREAST-Q scores					
Domain	Time point	Non-use (n = 14)	P-value	Use (n = 36)	P-value
		Median (IQR)		Median (IQR)	
Satisfaction With Breasts	Pre-op	56 (46–65)	0.906	53 (33–77)	0.111
	6 months	55 (46–96)		65 (54–85)	
Psychosocial Well-being	Pre-op	67 (60–75)	0.314	64 (54–91)	0.526
	6 months	79 (51–82)		75 (59–100)	
Sexual Well-being	Pre-op	54 (38–65)	0.779	54 (48–63)	0.497
	6 months	47 (32–80)		57 (45–68)	
Physical Well-being: Chest	Pre-op	85 (72–96)	0.172	81 (68–91)	0.708
	6 months	74 (67–88)		81 (70–93)	
Physical Well-being: Abdomen	Pre-op	83 (83–100)	0.128	83 (83–100)	0.949
	6 months	79 (61–89)		79 (79–92)	
Comparison of 12-month post-reconstruction with pre-op BREAST-Q scores					
Domain	Time point	Non-use (n = 14)	P-value	Use (n = 36)	P-value
		Median (IQR)		Median (IQR)	
Satisfaction With Breasts	Pre-op	56 (46–65)	0.102	53 (33–77)	0.068
	12 months	68 (54–85)		67 (50–80)	
Psychosocial Well-being	Pre-op	67 (60–75)	0.451	64 (54–91)	0.406
	12 months	75 (57–86)		79 (52–98)	
Sexual Well-being	Pre-op	54 (38–65)	0.906	54 (48–63)	0.889
	12 months	49 (39–66)		52 (38–72)	
Physical Well-being: Chest	Pre-op	85 (72–96)	0.161	81 (68–91)	0.699
	12 months	79 (65–91)		81 (68–91)	
Physical Well-being: Abdomen	Pre-op	83 (83–100)	0.001*	83 (83–100)	0.543
	12 months	79 (60–89)		89 (70–100)	

Neither group demonstrated significant within-group changes from the pre-operative to the 6-month post-final reconstruction time point (Table 6). The medication non-use group demonstrated a significant within-group decrease for Physical Well-being: Abdomen from the pre-operative to 12-month post-final reconstruction (median = 83, IQR = 83-100 versus median = 79, IQR = 60-89; p < 0.001). The medication use group did not demonstrate any significant within-group difference from the pre-operative to 12-month post-final reconstruction. Except for Sexual Well-being, all scores in the medication use group remained the same or increased from pre-operative baseline to 12 months after the final reconstruction. Only Satisfaction With Breasts and Psychosocial Well-being increased in the medication non-use group from the pre-operative baseline to 12 months after the final reconstruction.

Staged reconstruction patients’ scores decreased from the pre-operative time point to the 6-week post-TE placement time point across all domains. Scores at 6 and 12 months following final reconstruction were higher than the six-week post-TE placement time point scores for all patients. Figures 1 and 2 display the changes in scores across the four time points, between and within the two compared groups.

Sexual Well-being demonstrated statistically significantly lower within-group BREAST-Q scores among medication users in the depression group from the pre-operative to 6-week post-TE time points (median = 54, IQR = 48-63 versus median = 40, IQR = 20-49; p = 0.001) (Table 6). Anti-depressant medications were further categorized as those causing sexual dysfunction (n = 30, 83%) and non-dysfunction (n = 6, 17%) to perform a subgroup analysis in this domain. One time point demonstrated clinically significantly lower scores in the medication sexual dysfunction group: the six-week post-TE (median = 39, IQR = 26-47 versus median = 54; IQR = 0-63; delta = 15) time point (Table 7).

**Table 7:** Between-group comparisons of BREAST-Q Sexual Well-being scores for clinical significance [0.5 standard deviation (SD)] between patients using anti-depressant medications associated with (“Dysfunction”) and without (“Non-dysfunction”) sexual dysfunction at four time points. TE: tissue expander; IQR: interquartile range; SD: standard deviation; pre-op: pre-operative Mann-Whitney U test. Statistical significance was observed at p < 0.05; *statistically significant differences in comparisons. Clinically significant differences were >0.5 SD; +clinically significant differences in comparisons.

Time point	n	Dysfunction (n = 30)		n	Non-dysfunction (n = 6)		P-value
		Median (IQR)	0.5 SD		Median (IQR)	0.5 SD	
Pre-op	29	53 (46–63)	10	6	62 (54–77)	11	0.125
6 weeks post-TE	11	39 (26–47)	14	3	54 (0–63)	9	0.389^+^
6 months	18	57 (46–70)	10	3	54 (28–69)	11	0.762
12 months	29	52 (39–70)	15	6	55 (30–87)	13	0.759

## Discussion

In this study of PROs using the BREAST-Q, our results show that women who had a diagnosis of depression prior to a breast cancer diagnosis reported significantly lower Sexual Well-being scores than women with no diagnosis of depression at the six-week post-TE insertion time point. Because some anti-depressants are associated with sexual dysfunction, we subgrouped the anti-depressant medication users to determine if medication use disproportionately influenced the Sexual Well-being domain. Although we found no statistically significant differences, we found that patients using anti-depressants associated with sexual dysfunction had clinically significantly lower BREAST-Q scores at the six-week post-TE insertion time point for Sexual Well-being. These patients may benefit from additional counseling from healthcare professionals prior to and following TE insertion to help them progress to the six-month post-final reconstruction time point when their self-reported QOL is comparable to patients without depression or depressed patients using anti-depressants that do not cause sexual dysfunction.

This is an important consideration when assessing Sexual Well-being scores. Breast surgery may adversely change a woman’s perception of her own sexuality, leading to additional decreases in scores among women on anti-depressants associated with sexual dysfunction. A study by Mathias et al. (2006) evaluated the use of the anti-depressant bupropion, which is not associated with sexual dysfunction, on sexual function scores using the Arizona Sexual Experience Scale (ASEX) in women receiving adjuvant therapy for breast cancer. The use of this medication improved patients’ libido, sexual excitability, and ability to achieve orgasm [17]. In our study, six (17%) of the 36 patients with a prior diagnosis of depression were currently taking either bupropion or mirtazapine, two medications that have been shown to have minimal adverse effects on sexual function. As a post-hoc analysis, we evaluated BREAST-Q scores in patients on anti-depressants that did and did not cause sexual dysfunction to see if there were differences in their Sexual Well-being scores. Patients taking either bupropion or mirtazapine had clinically higher Sexual Well-being BREAST-Q scores at baseline compared to patients taking anti-depressants associated with sexual dysfunction (median = 62 versus median = 53). When assessing depressed patients who underwent staged reconstruction and stratifying by anti-depressant type, six-week post-TE insertion Sexual Well-being BREAST-Q scores were lower with anti-depressants causing sexual dysfunction (median = 39 versus median = 54).

Healthcare providers managing baseline depression may consider switching patients to an anti-depressant that does not cause sexual dysfunction or providing additional counseling for patients newly diagnosed with breast cancer. SSRIs are the most commonly prescribed anti-depressants with 56% of the 36 patients in our study taking an SSRI. Paroxetine is a commonly prescribed SSRI that inhibits the metabolism of the enzyme CYP2D6. This enzyme is necessary for the metabolism of the prodrug tamoxifen, a selective estrogen receptor modulator (SERM), commonly prescribed for breast cancer [18]. Preventing the conversion of tamoxifen to its active form can result in a limited effect, or even no therapeutic effect of the SERM. This may further provide an opportunity for healthcare providers managing depression to switch patients to anti-depressant medications that do not adversely affect sexual function, possibly benefitting the sexual well-being of women undergoing breast reconstruction. Additional research may prove to be beneficial.

One in five women will experience an episode of depression within their lifetime [19] The risk of depression and breast cancer both increase with age [6,20]. Originally, investigators believed there might be a correlation between depression, anti-depressants, and breast cancer. Studies have shown a psychological benefit to prescribing anti-depressant medications to women with breast cancer [21,22]. Depression is even more prevalent in patients diagnosed with cancer, which may influence QOL measures following breast reconstruction. Previous studies of PRO tools have evaluated the impact of depression and anxiety on different breast reconstruction options at different time points; however, these mood disorders were directly influenced by the underlying diagnosis of breast cancer [7,9,23].

One may assume that a patient’s QOL assessed by PRO tools may correlate with lower scores in patients with depression. However, a study by Salibasic et al. (2018) demonstrated no difference in a patient’s QOL scores before and after mastectomy in patients with or without significantly increased levels of depression, as measured by the European Organization for Research and Treatment of Cancer Quality of Life Questionnaires-Core30 and BR23 questionnaires [7]. While the BREAST-Q is a validated PRO measure, it is not known if PROs collected using the BREAST-Q may be influenced by anti-depressant use. For this reason, questionnaires validated to assess depression may provide more accurate assessments of patient QOL and satisfaction as they progress through the breast reconstruction process. Using more than one outcome tool concurrently with a history and physical examination may provide a more comprehensive approach to improve patient care [24,25].

This study has several limitations. Although the BREAST-Q data were collected prospectively, this study employed a retrospective analysis of data. BREAST-Q data for some domains and time points were incomplete as not all patients fully completed all questionnaires. The six-week time point was only collected for patients with staged tissue expansion. Due to our relatively small sample size, we included patients who underwent either TE-based or immediate autologous breast reconstruction; larger future studies should consider parsing out these groups to control for QOL differences relating to the timing of final breast reconstruction. Additionally, we categorized four patients with no prior diagnosis of depression who were taking an anti-depressant for another indication into the no depression group. Not all patients taking anti-depressants associated with sexual dysfunction were assessed clinically for symptomatic sexual dysfunction. Patients taking these anti-depressants may exhibit different adverse effects. In addition, sexual dysfunction may have been present throughout the entire study population secondary to anti-hormonal therapy for breast cancer, post-menopausal states, or psychologically from breast cancer or breast surgery. Finally, while we used non-parametric statistical analyses to report medians and IQRs, we assessed clinical significance and the MID using means and one-half of the SD, as previously described in the literature.

Larger multi-center studies are needed to further elucidate the role of a prior diagnosis of depression and anti-depressant medication use with PROs using the BREAST-Q questionnaire. Although the BREAST-Q has been validated, it has not been specifically tested in patients with mood disorders or who take medications for mood disorders. This may limit the utility of the BREAST-Q in certain populations. In addition, psychological interventions may be equal or more efficacious than anti-depressants alone in this population, as psychological interventions can be tailored to patients’ concerns (regarding body image, sexuality, etc.). Multimodal combinations of anti-depressants and psychological interventions may provide the best treatment strategy. Our findings provide a window to new information that may contribute to better patient care and the opportunity for future prospective studies.

## Conclusions

Patients with a pre-cancer diagnosis of depression had lower BREAST-Q Sexual Well-being scores at the six-week TE time point than patients without a diagnosis of depression. This was evident even for patients with depression on anti-depressant medications. Patients with a pre-cancer diagnosis of depression considering TEs may benefit from additional counseling prior to breast reconstruction or electing a different method of breast reconstruction. Anti-depressant medications may provide clinically higher BREAST-Q scores in patients with a pre-cancer diagnosis of depression. Adding an anti-depressant medication to a patient’s treatment plan may provide additional benefits.
