# Correction: The Effects of Depression and Anti-Depressants on Quality of Life After Breast Reconstruction: A Post-Hoc Analysis

**DOI:** 10.7759/cureus.c54

**Published:** 2021-12-08

**Authors:** Kevin M Klifto, Faraah N Bekheet, Michele A Manahan, Kristen P Broderick, Damon S Cooney, Gedge D Rosson, Carisa M Cooney

**Affiliations:** 1 Plastic and Reconstructive Surgery, University of Missouri, Columbia, USA; 2 Department of Plastic and Reconstructive Surgery, Johns Hopkins University School of Medicine, Baltimore, USA; 3 Department of Plastic and Reconstructive Surgery, Johns Hopkins Health System, Baltimore, USA

This article has been corrected to remove images of the BREAST-Q scales . Permission to use the BREAST-Q is granted by way of a license agreement, signed by the intended users. The license agreement prohibits the reproduction of the BREAST-Q in publications or any other public documents. The authors of this article violated the license agreement by reproducing the BREAST-Q scales in the Appendix of this article. As a result these images have now been removed.

